# Rates of Primary and Secondary Prevention of Cervical Cancer: A Study in a Province in the South of Italy

**DOI:** 10.3390/vaccines11111688

**Published:** 2023-11-03

**Authors:** Miriam Gorgone, Andrea Squeri, Sara Cuffari, Vincenza La Fauci, Ioselita Giunta, Serena Calderone, Raffaele Squeri, Cristina Genovese

**Affiliations:** 1 Department of Biomedical and Dental Sciences and Morpho Functional Imaging, Postgraduate Medical School of Preventive Medicine and Hygiene, University of Messina, 98121 Messina, Italy; miriam_gorgone@hotmail.it (M.G.); iosy511@gmail.com (I.G.); serenamariacalderone@gmail.com (S.C.); 2 Medical Oncology Unit, Department of Human Pathology “G. Barresi”, University of Messina, 98122 Messina, Italy; andreasqueri93@hotmail.it; 3 Local Health Unit of Messina, 98123 Messina, Italy; sara.cuffari@asp.messina.it; 4 Department of Biomedical and Dental Sciences and Morpho Functional Imaging, University of Messina, 98124 Messina, Italy; vlafauci@unime.it (V.L.F.); squeri@unime.it (R.S.)

**Keywords:** cervical cancer, HPV, vaccine, prevention, PAP test, screening, HPV-DNA

## Abstract

In Italy, cervical cancer represents the fifth most prevalent cancer in women under 50 years of age and is one of the most commonly detected lesions globally. Given the developing burden of the disease and the availability of both primary and secondary prevention measures, their accurate surveillance is of paramount importance. The aim of this study was to evaluate the trends in cervical cancer screening adherence in the period between 2020 and 2022, as well as to evaluate positive tests, identifying the most frequently associated genotypes and the vaccination coverage. The study sample was made up of 6880 women from the health district of Messina. We highlighted that there was a high proportion of positive results in the investigated period, with a high prevalence of HSIL. Moreover, HPV vaccination coverage was clearly inadequate, as was adherence to screening, both far away from WHO goals. This finding is probably linked to inadequate communication and awareness of the issue in the population and to the lack of data relating to tests carried out privately. In accordance with existing data in the literature, the introduction of the HPV-DNA test in Sicily made it possible to identify women positive for the genotypes most frequently involved in the etiopathogenesis of neoplastic lesions (genotypes 16 and 18), as well as for those in the “others” category, which should be investigated because some of them could have an impact on carcinogenicity and, for this reason, a future vaccine including them could represent a new prevention weapon.

## 1. Introduction

Cervical cancer is the second cancer most prevalent in the world, after breast one, with 500,000 new cases and 250,000 deaths every year [1]. In Italy, cervical cancer represents the fifth cancer most common in women under 50 years of age [2]. The natural history of the infection is strongly conditioned by the balance established between the host and virus. The early stages of all infections are asymptomatic and only 5–7% of them progress to related lesions (precancerous disease) [3]. Infection persistence is the “condictio sine qua non” for evolution towards carcinoma, with the risk of progression depending on the severity and the type of lesions. The long latency time between infection and carcinoma allows for secondary prevention through screening. In Italy, the new prevention vaccination plan 2023–2025 refers to WHO’s call to action aiming for “the elimination of cervical cancer as a globally public health problem”. It has historically been known that it is difficult to discern the subsite (cervix, body) of lethal uterine tumors. In Italy, the overall mortality for this cancer has decreased in the last two decades (−1.4% per year). Contrary to other types of cancer, mortality from this type of carcinoma has higher rates in Southern than in Northern Italy (+22%) [4].

In Sicily, the regional database of cancer registries observed, for the period of incidence considered (2007–2011), an average annual rate of 6.1 × 100,000, with about 160 new cases on average per year, while mortality data from the Register of Names of Regional Causes of Death (ReNCaM) record an average of about 30 deaths a year. The median 5-year survival is just under 70% [5]. A variety of scientific evidence demonstrates that cervical cancer screening, by means of cytological examination of cells from cervical smears or Papanicolaou tests (PAP), prevents death from this disease. The recommended interval between tests is once every three years for the target population aged 24–65 years [6]. In Sicily, efforts made in recent years by the regional health system have made it possible to quadruple the number of cervical cancer screenings. However, in the two-year period 2020–202, data from the PASSI surveillance system show that the rate of early diagnosis is lower than that found in other areas of the country, as already has been reported in the previous period (71.9% vs. national average of 77.5%) [7].

In particular, in the Sicilian Local Health Unit (LHU), the last regional report on the percentage of women who reported having carried out a preventive PAP test in the last 3 years found it was around 69% (lower than the national reference average, which is 79%) [8]. In 2015, the National Screening Observatory (ONS), jointly with the Italian Cervical Carcinoma Screening Group (GISCI) and the main scientific societies in this area, promoted a consensus conference to define cervical screening pathways for women vaccinated against HPV. The indications provided by the consensus document (based on an ONS report dated 26 April 2021), based on the best scientific evidence, call for a modification of the screening protocol upon the arrival of cohorts of vaccinated girls (defined as those who received at least two doses by the end of their 15th year of age) as they carry a very low risk of pathology. These indications foresee the raising of the age of first invitation to a screening test to 30 years and identify the HPV test as a primary screening test [9]. In recent years, several studies demonstrated that cervical screening based on validated DNA tests for oncogenic types of HPV (HPV-DNA testing) is more effective than that based on cytology in preventing invasive cervical cancers [10]. The HPV-DNA tests currently used in diagnostics are based on detecting the DNA of various HPV types in clinical samples; in the context of screening protocols, it is sufficient to test the whole high-risk HPV group, without strain distinction. So, the term “HPV-DNA test” in screening only means looking for high-risk HPV types. The results of experimental efficacy studies have shown that the HPV-DNA screening test has greater sensitivity than the PAP test, but a lower specificity, and since HPV-positive cases are sent directly to colposcopy, this would lead to a high increase in colposcopies and a decrease in the positive predictive value (PPV) of the first-level test [11]. The introduction of a filter cytological examination between the high-oncogenic-risk HPV-DNA test and colposcopy would allow specificity to be brought back to acceptable values. The rationale is to overturn the current “PAP test followed by triage HPV test” algorithm, performing the most sensitive test first (HPV-DNA test) and then, if necessary, the most specific test (PAP test). In this context, the HPV-DNA test identifies women at risk of developing the disease and the PAP test identifies those infections in which the virus has begun to produce cellular alterations. In this way, the introduction of the HPV-DNA test has been shown, in experimental studies, to offer 60–70% higher protection against invasive cancer compared to the PAP test, and the screening interval can increase from 3 to 5 years [11]. 

By applying the correct protocol, the overall costs of screening based on the HPV-DNA test are estimated to be lower than those of conventional cytological screening with a 3-year interval, even if the cost per single round of screening is higher. For these reasons, the Italian 2014–2018 National Prevention Plan provided that by 2018, all regions would switch to the HPV-DNA test as a primary screening test [12]. The HPV test is, therefore, nowadays recommendable as a primary screening tool, if it is offered to women of at least 30 years of age, if appropriate protocols are applied and if HPV tests validated for the purpose are used. Indeed, HPV infection is frequent, has a prevalence peak at the onset of sexual activity and decreases with increasing age, regressing spontaneously in most cases; the persistence of infection is necessary for the development of intraepithelial lesions, which will occur only in some individuals (the presence of infection, therefore, does not imply progression towards a precancerous lesion). HPV testing is recommended for women aged 30 to 65 and should be performed every 5 years. The HPV test can identify women at high risk of developing cancer. Therefore, the examination should be reserved for women over the age of 30, since before this age, HPV infections are very frequent, and they spontaneously regress in a high percentage of cases. It must be made clear that (a) a positive HPV test does not necessarily equate to a cancer diagnosis; (b) the HPV test can be performed at longer intervals (at least five years) than the three years required for the PAP test; (c) the HPV test for early detection of cervical cancer should not be performed before the age of 30–35, for the above-mentioned reason.

Moreover, a descriptive study of hospital discharge records (HDRs) was conducted in Italy between 2008 and 2018. Overall, 670,367 hospitalizations due to HPV-related diseases occurred among Italian subjects. In addition, a significant decrease in hospitalization rates for several types of cancer such as cervical, vulval, vaginal and oropharyngeal cancer, as well as genital warts, was observed. This indicated the positive impact of HPV vaccination coverage and cervical cancer screening on hospitalizations due to cervical cancer [13,14]. 

The aim of this study was the evaluation of women’s adherence to screening during and after the pandemic period, testing the local rate of positive tests, identifying the most frequently associated genotypes and, at the same time, the evaluation of vaccination coverage, with particular attention to the onset of L-SIL or H-SIL after the immunization program in the cohort of patients born after 1996 (year of introduction of the HPV vaccine in the Sicilian region).

## 2. Materials and Methods

**Figure 1:**
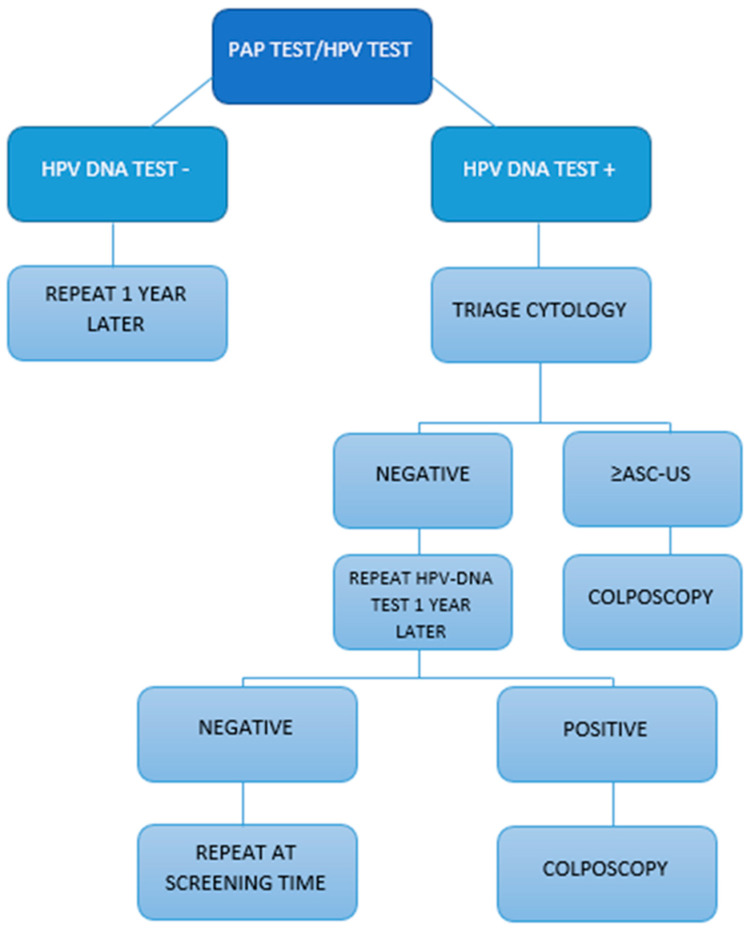
Screening protocol in the Sicilian region.

This retrospective study was conducted thanks to cooperation between “Universitary hospital G. Martino” (Hospital Hygiene Unit) in Messina and the Screening Management Center of the LHU in Messina, in the period from January to July 2023. The sample consisted of 6880 women aged between 25 and 64 years who had been contacted for cervical cancer screening according to the guidelines of the Sicilian region [15]. In detail, the women were screened over the three-year period January 2020–December 2022 (after an active call or home invitation by the Messina LHU) by means of a PAP test or HPV-DNA. A subsample was selected from this cohort; it was made up of women who continued the diagnostic process with a 2nd level investigation, totaling 110 women in 2021 and 174 women in 2022. The frequency of the various types of lesions and their possible correlation with high-risk genotypes and vaccination status were evaluated in this subsample. Screening data were collected through the Arianna software 1.0, while vaccination coverage through the On Vac software 4.31. For screening, we collected data on both types of tests, as required by the protocol of the Sicilian region: PAP test every three years for women aged between 25 and 33 and, eventually, HPV-DNA for triage in the presence of ASC-US cytology; the HPV-DNA test (introduced in the region with an assessorial decree on 08/2017) must be repeated as the primary test every five years for women aged 34–64. The screening protocol is shown in Figure 1.

Nowadays, 2 types of HPV-DNA tests are available: low- and high-risk (HR) HPV-DNA test. The former is a test that allows you to identify each high-risk (16, 18, 26, 31, 33, 35, 39, 45, 51, 53, 56, 58, 59, 66, 68, 69, 73 and 82) and low-risk (6, 11, 40, 42, 43, 44, 54, 61 and 70) HPV virotype. It is recommended in (a) women aged 30–35 every 3–5 years; (b) after a positive HR HPV-DNA test; (c) in the presence of cytologic abnormalities associated with low-risk virotypes. The latter is used for the exact identification of high-risk genotypes (16 and 18, from which 70% of cervical carcinomas are derived) and for generic screening for twelve high-risk virotypes (31, 33, 35, 39, 45, 51, 52, 56, 58, 59, 66 and 68). The type used in the Sicilian region is the latter, and so the results of our investigation could be positive/negative for genotypes 16 and 18 or positive/negative for high-risk types.

Continuous variables were expressed as mean ± standard deviation; categorical variables were expressed as absolute and percentage frequencies. Statistical analysis was performed with R software.

## 3. Results

From the data analysis, it appears that the increase in the effectiveness of cervical screening in the province of Messina (percentage of women aged 25–64 from the target population who received a letter of invitation from the year 2020 to 2022) was equal to 28% in the investigated period (140,784 women between 25 and 64 years received the invitation in the analyzed three-year period).

**Figure 2:**
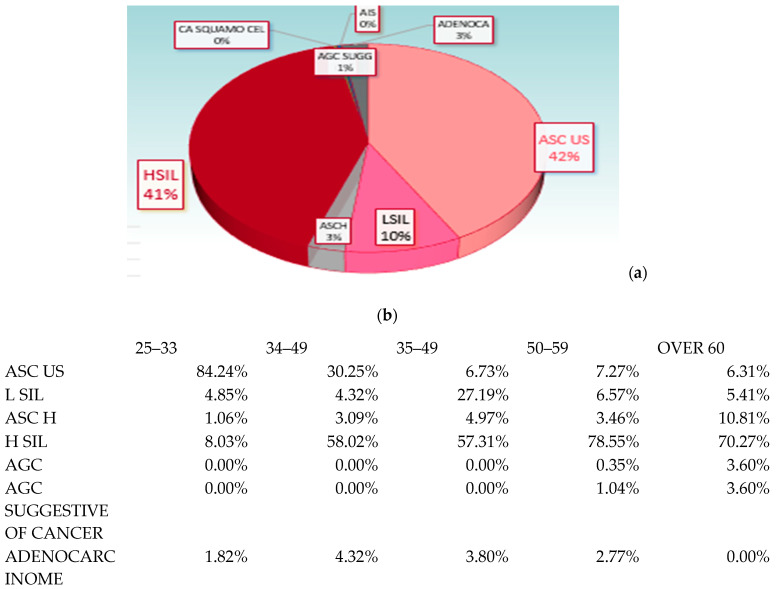
Frequency of various types of cytologic abnormalities in total (**a**) and by age group (**b**).

In the sample (n = 6880 women), the rate of adherence to screening in the province of Messina was evaluated in the three-year period under examination. In 2020, the compliance rate was 12.32%, with a growing trend in subsequent years, rising to a 16.85% adherence rate in 2022. In the sample investigated, 23% of screening test results were positive (specifically, 13% in the HPV-DNA test sample and 10% in the PAP test sample were positive) with different distributions in the various age groups and, in particular, with a higher prevalence in the 25–33 age range (41% in the 25–33 age group, 33% in the 35–49 group, 19% in the 50–59 group and 7% in the over-60 group) (Figure 2).

The most prevalent lesions in our sample were HSIL (respectively, 8.6% in 2020, 9.57% in 2021 and 14.45% in 2022), and so we recorded an increasing trend compared to previous years.

**Figure 3:**
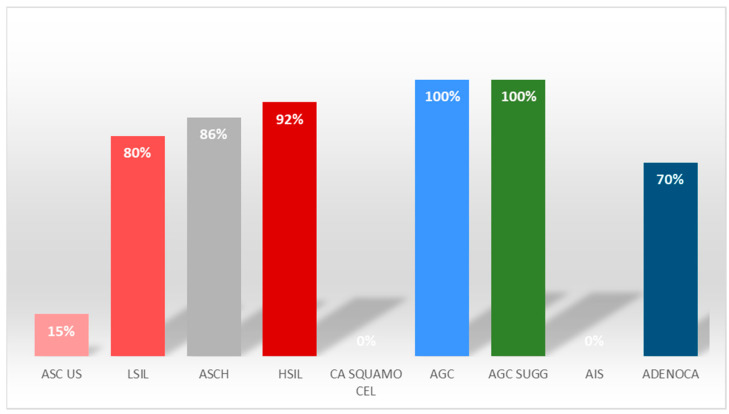
Distribution of positive HPV-DNA tests in different types of lesion in the period 2020–2022. Legend: ASC-US—atypical squamous cells of undetermined significance; LG-SIL or LSIL—low-grade squamous intraepithelial lesion; ASC-H—atypical squamous cells, cannot exclude HSIL; HGSIL or HSIL—high-grade squamous intraepithelial lesion; squamocellular cancer; AGC or AGC-NOS—atypical glandular cells not otherwise specified; AIS—endocervical adenocarcinoma in situ; ADENOCA—adenocarcinoma.

In addition, the rate of positive HPV-DNA test results by type of lesion was analyzed (see Figure 3). 

From the total number of women screened with both PAP tests and HPV-DNA, we then extrapolated and further analyzed the sample of women who continued the diagnostic process with a second-level investigation; specifically, we analyzed a sample of 110 women screened in 2021 (average age 34.4 ± 9.6 SD) and 174 in 2022 (mean age 39.3 ± 9.2 SD).

**Figure 4:**
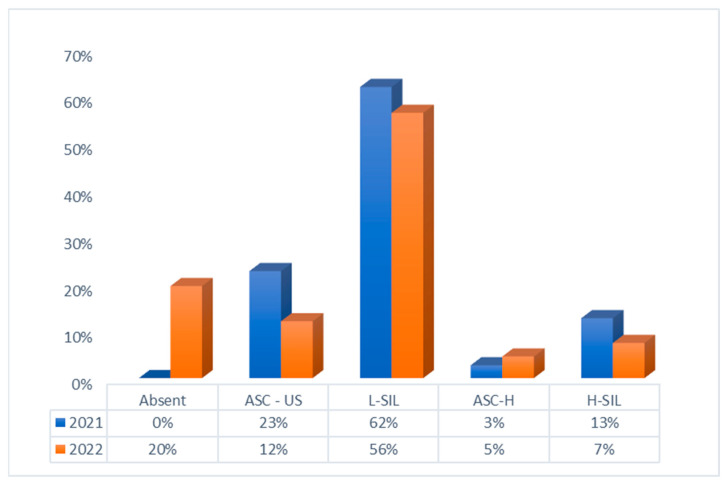
Type of PAP test results (frequency) per year.

In this cohort, we evaluated the frequency of the various lesion types and their possible correlation with high-risk genotypes by year. The results are shown in Figure 4.

**Figure 5:**
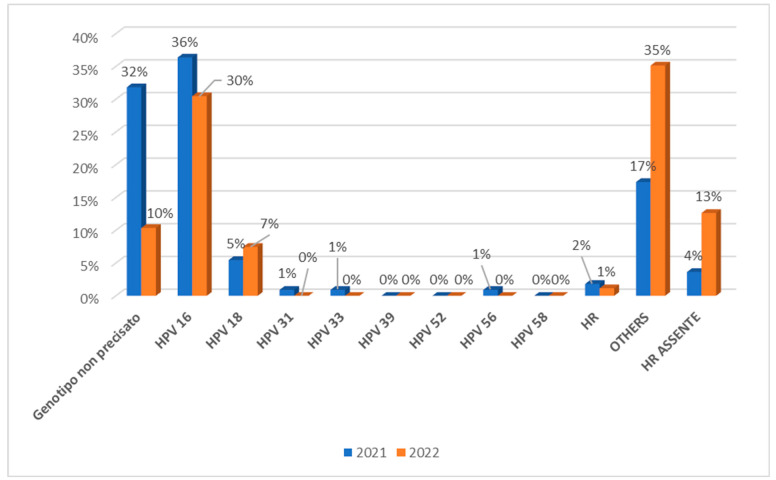
Isolated genotypes.

Among the genotypes identified, the most prevalent one was 16 (36% in 2021 and 30.45% in 2022), followed by HPV 18 (7.5%). In the remaining part of cases, other high-risk strains were found in 36.2% of cases, and other low-risk genotypes in 12.6% of cases (Figure 5).

Colposcopy with subsequent biopsy and histological examination, carried out as a confirmatory investigation, was negative for the presence of lesions in 19% and 20% of cases in 2021 and 2022, respectively, while in the remaining cases, a high-grade lesion (CIN3) was found in 12% of the 2021 cohort and in 14% of that of 2022; an intermediate-grade lesion (CIN2) in 14% (2021) and 22% (2022); and, finally, a low-grade lesion in 45% (in 2021) and 39% (2022) cases (CIN1). Only in 1% of the cases did we find cervical cancer.

The HPV 16 genotype was not only one of the most frequently isolated, but also the one associated with the most severe histological lesions.

Moreover, a high number of lesions (CIN1-3) associated with the “Others” category of genotype was found both in 2021 and 2022, with percentages ranging from 1% to 8% in 2021 and from 2% to 18% in 2022. 

We then analyzed data on vaccination coverage in the two cohorts examined by evaluating the percentage of subjects vaccinated at the time of diagnosis. We recorded that the rate of vaccinated people was 9% in 2021 (30% of this sample received the tetravalent vaccine) and 27% in 2022 (17% vaccinated with tetravalent vaccine).

Regarding the number of doses received at the time of diagnosis, it should be emphasized that the majority of subjects examined had received three doses (60% in the 2021 cohort and 57% in the 2022 cohort, respectively), while age at vaccination was less than 35 years in 80% of subjects examined in 2021 and in 55% of subjects in 2022, respectively (>35 years—20% in 2021 and 45% in 2022).

## 4. Discussion

In our study, data analysis showed that the adherence of cervical screening in the province of Messina was equal to 28% in the investigated period; this figure is markedly lower than that for the rest of the South (68.8%) and, unfortunately, still far from that for regions in Northern Italy [14,15,16]. 

Furthermore, the adherence data collected highlighted a poor adherence to HPV screenings, probably linked to multiple factors, one of which, at least initially, was the COVID-19 pandemic. In fact, as can be seen from the results presented, there was an upward trend in 2022 compared to the previous two years.

The low adherence rate that we found (12–16.85%) differs from the WHO recommendations, which require that 70% of women worldwide be routinely screened for cervical disease with a high-performance test and that 90% of those who need it receive adequate care. Indeed, together with vaccinating girls against human papillomavirus (HPV), the implementation of this comprehensive strategy could prevent more than 62 million deaths from cervical cancer in the next 100 years [17]. 

According to the WHO [18], moreover, the recommendations on vaccination and screening must undergo changes. Italy has already begun to implement them: the first change concerns the initial screening age and the type of screening test for girls vaccinated at 12 years old. As indicated by the Italian Consensus Conference and confirmed by the study “*Integration of vaccination and screening programs for the prevention of cervical cancer: interventions to redefine and implement new screening protocols for women vaccinated before the screening start age*” funded by the National Center for the Control of Infectious Disease (CCM) 2016 program, the initial screening age will be raised to 30 years with the introduction of HPV tests. In most regions, there were cohorts of women vaccinated at 12 who reached the age of 25 already in 2021. In other regions, initial screenings of cohorts vaccinated at 12 will take place in 2022. Based on the 2020–2025 National Prevention Plan, regions have the mandate to prepare specific “Free Programs” as regards cancer screening, one of which in particular relates precisely to the screening of vaccinated cohorts. Vaccination status is defined by matching the names of women invited for screenings with regional or corporate archives of vaccinated women. As demonstrated by the 2016 CCM study, this may be incomplete: for example, girls who resided in other regions or provinces are not vaccinated. 

In our study, the positivity rate for both types of test was 23% and, in particular, 13% were positive for the HPV test; this is a worrying value in light of the fact that HPV prevalence in the general population in Italy has a lower rate (8%) [19]. In fact, a systematic review of studies conducted in Italy found this value in the general population without substantial differences between the South, Center and North. It should be noted that the calculation of this prevalence only considered studies with a random sample of the population; studies recruiting women who present spontaneously to an ob-gyn clinic generally report higher prevalence estimates than studies recruiting women based on active invitation from the general population. This is probably because women who spontaneously access gynecological clinics have, in a variable proportion, previous positive PAP tests. These data are, therefore, worth paying attention to, since in our study we only considered active invitations.

In our sample, the prevalent lesion was HSIL; an increase in HPV prevalence was found both in the total sample and in women with abnormal cytology. In particular, in the examined three-year period, we recorded a prevalence of 15% in the case of ASCUS (atypical squamous cells of undetermined significance), 80% in the case of LSIL (low-grade lesions of the squamous intraepithelial cells), 86% in the case of ASC-H, 92% in the case of HSIL (high-grade lesions of the squamous intraepithelial) and 70% in the case of adenocarcinoma. 

Biopsy analysis allowed our study to evaluate the genotypes most implicated in the various types of lesions; first, according to the data collected at a national and global level, the genotype most frequently associated with the lesions was HPV 16; subsequently, in contrast to nationally conducted studies, in second place we found the “Others” category and not HPV 18. In fact, type-specific prevalence studies in Italy pointed out that the most frequent type of virus is HPV 16. The prevalence of HPV 16 in the healthy population is approximately 5% (2–10%), while the prevalence of HPV 18 is lower, with an average value of just over 1% (0–6%).

In our study, we also evaluated the prevalence of the various types of HPV in cytological lesions in the two-year period 2021–2022. As can be seen in Figure 4, province-level findings were positive for the most severe dysplasia (CIN 3) in about 18% of cases; in 12% for CIN2; and in about 10% for CIN 1. These data are different from national-level data, where the national average of HPV 16 positivity is 35% in milder dysplasia, 64% in more severe dysplasia and 68% in invasive carcinomas [20].

It is important to underline that, in the period under study, there was a fair number of lesions, even of an advanced degree, associated with the “others” genotype category, supporting the implication of these genotypes and hence the need to test more and to create a provincial dataset to evaluate their prevalence in our city. 

Finally, we wanted to evaluate the percentage of vaccinations in screened women in the subsample: the percentage of vaccinated women in 2022 was 27%; in 80% of cases, the age at vaccination was less than 35 years, with a cycle of at least two doses only found in 20% of the sample [21].

This figure is also important in light of recent scientific evidence which demonstrated that HPV vaccination was associated with complete viral elimination in 86% of cases. Analyzing the differences between healed and unhealed, it was highlighted that the vaccinated, while producing anti-HPV antibodies, had a significantly lower antibody rate, about seven times, compared to the healed. 

The study states that the time to virus elimination for people who already have the infection is, on average, about two years, in the meantime exposing these subjects to the risk of infertility, multiple miscarriages, condylotomies and HPV-related tumors. In the subjects investigated, vaccination proved to be able to induce the elimination of the virus in 86% of cases, through an increase in the rate of circulating anti-HPV antibodies. They also highlighted how a cut-off value equal to or greater than the threshold value of 1:125, obtained from the analysis of the ROC curve, can determine a greater clearance rate, thus confirming the therapeutic efficacy of the vaccine. Therefore, in cases of low antibody production, it is possible to hypothesize the use of a second vaccination cycle or an additional dose to obtain a level of antibodies capable of guaranteeing the curative effect.

In conclusion, we can state that the analysis of the results brought out an inadequate adherence rate, certainly far from the values present in other situations in Northern and Central Italy, confirming the data present in the literature, and far too low to achieve the elimination of cervical cancer by 2030.

This finding is probably linked to inadequate communication and awareness of the issue of screening in the population. Another determining factor could be represented by under-reporting relating to the number of screening tests carried out privately.

From this perspective, possible strategies to combat this issue could be to create a synergy between the local authorities and gynecologists, to recover data relating to the remaining portion of the population that presumably performs the tests privately, to involve GPs (General Practitioners) as a tool for action in light of the relationship of trust established with patients and finally to strengthen educational and communication campaigns. Finally, it is clear that the COVID-19 pandemic certainly impacted adherence to screenings, due to the fear of contagion that the global population experienced in the three-year period under study, the loss of efficiency in routine activities due to dealing with the emergency as well as due to the initial restrictive measures.

Self-sampling could, in such cases, represent an essential tool in overcoming possible difficulties in accessing the screening sampling clinics (hindered, for example, by lack of time, modesty/shame or religious motivations). Self-collection has already been proposed in various European countries [22] and in developing countries. In Italy, it has been proposed as part of research projects to thousands of women residing in Tuscany, Veneto, Abruzzo and Lazio [23,24,25] and as part of a pilot project to women residing in Umbria and Tuscany and, finally, in some screening programs (for example, in some programs in Veneto and Emilia-Romagna). 

A strength of our study is certainly its retrospective nature, which allowed us to record real and unreported data, unlike other types of studies present in the literature; however, the limitation linked to the lack of data from privately performed screenings and then the impossibility of further analyzing all HPV genotypes present in the population under examination should be noted.

## 5. Conclusions

Finally, we can state that many interventions could be useful for increasing not only screening uptake but also vaccine coverage, especially for at-risk categories such as pregnant women, frail patients and healthcare workers. This last category has a fundamental role in fighting vaccine hesitancy and more interventions must be implemented to increase the knowledge and expertise of many categories of HCWs in terms of vaccine use in general population. Public health specialists are the main figures driving and serving as an example to increase prevention strategies [26,27,28,29]. 

## Data Availability

Data available on request from the authors.
